# Work accidents with children and youth in a rural environment in southern Brazil

**DOI:** 10.1590/1518-8345.3188.3243

**Published:** 2020-02-03

**Authors:** Daiani Modernel Xavier, Marta Regina Cezar-Vaz, Clarice Alves Bonow, Maria Denise Schimith

**Affiliations:** 1 Universidade Federal do Rio Grande/FURG, Escola de Enfermagem, Rio Grande, RS, Brazil.; 2 Universidade Federal de Pelotas/UFPel, Departamento de Enfermagem, Pelotas, RS, Brazil.; 3 Universidade Federal de Santa Maria, Departamento de Enfermagem, Santa Maria/UFSM, RS, Brazil.

**Keywords:** Accidents, Occupational, Child, Adolescent, Family, Rural Population, Nursing, Acidentes de Trabalho, Criança, Adolescente, Família, População Rural, Enfermagem, Accidentes de Trabajo, Niño, Adolescente, Familia, Población Rural, Enfermería

## Abstract

**Objective:**

to know the prevalence of occupational accidents in children and youth who work with their families in the rural environment and to identify the associated factors.

**Method:**

exploratory, descriptive and analytical study with quantitative approach, developed in three rural areas. Participants were 211 children and young people who assisted the family in rural work. Data collection was performed using a semi-structured questionnaire. Bivariate analysis was performed using Pearson’s chi-square, Fisher’s exact, Student’s t and Mann-Whitney tests and multivariate analysis using Poisson regression.

**Results:**

the prevalence of self-reported occupational accidents was 55%. It was highlighted: insect bites (44%), burns (40.5%), falls (27.6%), injury with a working tool (16.4%), electric shock (15.5 %), burn by animal (8.6%), animal bite (6.9%) and pesticide poisoning (2.6%). These were related to shared housing, leisure activity - riding a motorcycle, product resulting from lettuce cultivation and use of personal protective equipment.

**Conclusion:**

it is believed that these findings may enhance the development of public policies aimed at preserving the health of these children and young people, regulate working conditions and reduce occupational risks in the rural environment.

## Abstract (pt)

**Objetivo:**

conhecer a prevalência de acidentes de trabalho em crianças e jovens que trabalham com a família no ambiente rural e identificar os fatores associados.

**Método:**

estudo exploratório, descritivo e analítico, com abordagem quantitativa, desenvolvido em três ambientes rurais. Participaram 211 crianças e jovens que auxiliavam a família no trabalho rural. A coleta de dados foi realizada por meio de questionário semiestruturado. A análise bivariada foi realizada utilizando-se os testes qui-quadrado de Pearson, exato de Fisher, t de Student e Mann-Whitney e análise multivariada, por meio da regressão de Poisson.

**Resultados:**

a prevalência de acidentes de trabalho autorreferidos foi de 55%. Destacaram-se: picadas de insetos (44%), queimaduras (40,5%), quedas no ambiente de trabalho (27,6%), lesão com instrumento de trabalho (16,4%), choque elétrico (15,5%), queimadura por animais (8,6%), mordida de animais (6,9%) e intoxicação por uso de agrotóxicos (2,6%). Esses relacionaram-se com moradia mista, atividade de lazer - andar de motocicleta, produto resultante do cultivo de alface e uso de equipamentos de proteção individual.

**Conclusão:**

acredita-se que esses achados possam incrementar o desenvolvimento de políticas públicas direcionadas à manutenção da saúde dessas crianças e jovens, ao controle das condições de trabalho e à redução dos riscos ocupacionais no ambiente rural.

## Abstract (es)

**Objetivo:**

conocer la prevalencia de accidentes de trabajo en niños y jóvenes que trabajan con la familia en ambiente rural e identificar los factores asociados.

**Método:**

estudio exploratorio, descriptivo y analítico, con abordaje cuantitativo, desarrollado en tres ambientes rurales. Participaron 211 niños y jóvenes que auxiliaban a la familia en el trabajo rural. La recolección de datos fue realizada por medio de cuestionario semiestructurado. El análisis bivariado fue realizado utilizando las pruebas: Chi-cuadrado de Pearson, exacta de Fisher, t de Student y Mann-Whitney y el análisis multivariado, por medio de la regresión de Poisson.

**Resultados:**

la prevalencia de accidentes de trabajo auto relatados fue de 55%. Se destacaron: picadas de insectos (44%), quemaduras (40,5%), caídas en el ambiente de trabajo (27,6%), lesión con instrumento de trabajo (16,4%), choque eléctrico (15,5%), quemadura por animales (8,6%), mordida de animales (6,9%) e intoxicación por uso de pesticidas (2,6%). Esos se relacionaron con vivienda mixta, actividad de ocio (andar de motocicleta), cultivo de lechuga y uso de equipamientos de protección individual.

**Conclusión:**

se piensa que esos hallazgos podrían incrementar el desarrollo de políticas públicas dirigidas a la manutención de la salud de esos niños y jóvenes, al control de las condiciones de trabajo y a la reducción de los riesgos ocupacionales en el ambiente rural.

## Introduction

Individuals living in rural environment are exposed to socioeconomic vulnerabilities that force them to develop subsistence work activities from an early age. Often, these activities are carried out within the family and involve the help of children and youth^(^ 1 ^)^. In this context, they generally work in subsistence agriculture, fisheries and cattle, performing tasks such as soil preparation, crop planting, harvesting, loading of products, preparation of fishing nets and the raising of cattle, horses and sheep^(^ 2 ^)^. It is noteworthy that the inclusion of these children and youth in rural work can be positive for their civic development, as well as for the family economy if it does not, negatively, affect health and/or education^(^ 3 ^)^.

In carrying out their activities to help relatives in rural work, children and young people often use and/or are exposed to rivers, deep seas and lakes, animals, chemicals, dangerous machinery and tools, heavy lifting and transportation of products through automotive vehicles such as tractors and trucks. In addition, there are the long periods of time in these tasks, which require attention and muscle strength, fast pace of production, staying in awkward body positions and the different weather conditions they face, such as extreme temperatures and storms that put them at risk of accidents during work^(^ 2 ^,^ 4 ^-^ 5 ^)^.

National and international evidence points that accidents in rural work, especially in the agriculture and fisheries subcategories, represent a major cause of accidents and mortality in children and young people of both sexes, aged 10 to 24 years^(^ 6 ^)^. Drowning, traffic accidents, burns, falls, contusion, fractures, transfixing injuries and crushing as common events resulting from inattention during work due to physical and mental exhaustion, caused by complex and dangerous tasks, are highlighted in this age group^(^ 1 ^,^ 4 ^,^ 6 ^-^ 8 ^)^.

Although the inclusion of children and young people in agricultural, fishing and cattle raising activities in rural family communities is advantageous from the subsistence point of view, it can represent an obstacle to their biopsychosocial development. Still, it can be detrimental to the health and safety of these individuals, since they are often unaware of the risks to which they are exposed and assume activities beyond their physical and mental capacities, which favors the occurrence of occupational accidents^(^ 1 ^-^ 2 ^)^.

In this perspective, alternatives are needed to take these children and young people off from the vulnerability and risks which they are at. International literature emphasizes that public policy investment aimed at accessing education in rural communities has the potential to generate economic benefits for these individuals and to public finances. Moreover, quality education can stop the vicious cycle of poverty, typical of the rural economy^(^ 2 ^)^.

In addition, children and young people living and working in rural areas need to be a priority in health actions. Nurses are professionals who work directly with individuals in the context of collective health, predominantly in primary health care. In this sense, their cooperation with the provision of care to children and young people exposed to the risks of work in their productive processes is essential, along with the production of scientific and empirical knowledge that grounds public policies, focusing on protection, surveillance, health promotion and disease prevention to this public. Thus, investigating the health-work process relationship, in the focus of occupational accidents in rural environments, represents an important contribution in the area of ​​collective health and occupational nursing.

Given the above, the guiding question of this study was: what are the occupational accidents that affect children and young people who work with their families in the rural environment and the most frequent associated factors? From this question, the objective is to know the prevalence of occupational accidents in children and youth who work with their families in the rural environment and to identify the associated factors.

## Method

This is an exploratory, descriptive and analytical study with a quantitative approach. It was developed in three rural environments in southern Brazil. The participants were 211 children and young people exposed to risks and accidents at work in these places, during the study period.

Due to the lack of records of the total number of children and young workers in rural areas in official sources, state and municipal agencies made an estimate based on the percentage of the population, according to age groups (10-14 which is equivalent to 4.4%, 15-17 equivalent to 4.5% and 18-24 equivalent to 4.5%), according to the distribution described in the Brazilian Institute of Geography and Statistics^(^ 9 ^)^. Based on this criterion, these percentages of the general population were applied to the total inhabitants of each rural environment, that is, the first (1,800 inhabitants), the second (1,200 inhabitants) and the third (400 inhabitants). Of this total of 3,400 inhabitants, 456 corresponded to the age group surveyed. Subsequently, the total number of the sample was calculated using the Stat Calc tool of the Epi Info 6.04 program, with a 95% confidence level, resulting in a minimum number of 209 children and young people aged 10 to 24 years. However, a total of 211 participants were reached.

The sample selection was based on the following inclusion criteria: being resident in rural areas of the municipality included in the research, having a minimum age of 10 and a maximum of 24 years and working with the family in the rural environment, with or without periodic remuneration. This age group was established according to the epidemiological bulletin, developed by the Integrated Program on Environmental and Workers Health (Pisat), in partnership with the Collaborating Center for Surveillance of Occupational Health Disorders of the Ministry of Health, which showed high rates of cases of occupational accidents and mortality in children and young people in this age range^(^ 6 ^)^.

Data collection was performed by the main researcher, at the house of each participant by prior appointment, from January to March 2018. A semi-structured questionnaire with the participants’ sociodemographic characteristics (age, gender, skin color, marital status, level of education, type of housing with regard to constitution and property and family income), rural working environment (work performed, work activities, main products resulting from work activity, working time, daily working hours, leisure activities, leisure hours), the use of Personal Protective Equipment (PPE) and reasons for not using them (respirator mask, waterproof coat and pants, face visor, arabic hat, rubber boots, waterproof gloves, helmet, mask, brimmed hat, ear protectors and sunscreen) and the occurrence and reasons of work accidents was applied. This questionnaire was made based on a previous reference used in previous studies of the Laboratory of Socio-Environmental Studies and Collective Health Production (Lamsa)^(^ 10 ^-^ 11 ^)^.

A pilot test was conducted with ten children and young people in a rural environment, prior to data collection, in order to confirm the validity of the instrument, that is, to identify the need for semantic modifications in the questions and to verify if the research design would make it possible to achieve the proposed objectives. Due to no need for adjustments regarding nomenclatures, as well as in the detail and clarity of the questions, the answers of the questionnaires were included in the study.

The collected data were entered in Epi Info software, version 6.04, with double typing, to refine them. For descriptive statistical analysis, data were transported to the Statistical Package for Social Science (SPSS) software, version 21.0.

Quantitative variables were described by mean and standard deviation or median and interquartile range. Student’s t-test for independent samples was used to compare the means between children and young people who suffered work accidents and those who did not, which allowed the binary categorization of this outcome and subsequent association with the other variables described above. To evaluate symmetry, the Shapiro-Wilk test was used, together with the magnitude of the standard deviation. In case of asymmetry, the Mann-Whitney test was used.

Categorical variables were described by absolute and relative frequencies. In the comparison of proportions, Pearson’s chi-square or Fisher’s exact tests were used. To control confounding factors, multivariate Poisson regression analysis was applied. The criterion for entry of the variable in the model was p<0.20 in the bivariate analysis, and the criterion for its permanence in the model was to remain at a p value <0.10 in the final model. The adopted significance level was 5% (p≤0.05).

Ethical principles were respected, according to Resolution 466 of December 12^th^, 2012 on research involving human beings^(^ 12 ^)^. The research project was approved by the Ethics Committee of the Federal University of Rio Grande (FURG), under Opinion n. 113/2017. An Informed Consent Form (ICF) were given to participants, which provided clarifications on the study, inviting them, explaining the objectives and proposed methodology and requesting their consent to participate in the study, when older than 18 years. For lower ages, the consent form was signed by both, the child or youth and their legal guardian, ensuring respect for the ethical aspects involved in the research, such as the right to privacy and the anonymity of the participants.

## Results

The sample of this study consisted of 211 children and young people. Regarding their sociodemographic data, 118 (55.9%) were female, 190 (93%) single, 150 (75.6%) white and 115 (54.5%) had incomplete elementary school. The participants’ ages ranged from 10 to 24 years, with an average of 15 years. Regarding the work characterization, they helped the family, especially in activities alluding to agriculture and fishing, for four daily hours and average working time of three years.

Of the total participants, 116 (55%) self-reported some type of occupational accident. It was highlighted: insect bites (44%) such as bee (11.8%) and mosquito (11.4%), burns (40.5%), falls in the workplace (27.6%), injury with working tool (16.4%), electric shock (15.5%), burn by animals (8.6%), animal bite (6.9%) and pesticide poisoning (2.6%).

Among the causes reported by children and young people for occupational accidents were: lack of attention at work (93.7%), lack of technical knowledge (5.2%), lack of protective equipment at work (3.4%), work overload (1.7%) and too many parallel activities (0.9%). Because minority of children and young people (95; 45%) did not report occupational accidents, this outcome was qualified in the participant’s own reference when expressing the occupational accident as follows: with an occupational accident or without an occupational accident. After binary categorization of the outcome, it was associated with the other variables under study, which are presented in Table 1.

**Table 1:** Association of sociodemographic variables under study with occupational accidents (n=211). South Region, Brazil, 2018

Variables*	Total Sample (n=211)	With occupational accident (n=116)	Without occupational accident (n=95)	P value
Age (years)	15.2±2.9	15.0±3.2	15.5±2.4	0.198^†^
Gender				1.000^‡^
Male	93 (44.1)	51 (44.0)	42 (44.2)	
Female	118 (55.9)	65 (56.0)	53 (55.8)	
Color				0.320^‡^
White	158 (75.6)	94 (81.0)	64 (68.8)	
Black	19 (9.1)	9 (7.8)	10 (10.8)	
Brown	23 (11.0)	9 (7.8)	14 (15.1)	
Indigenous	6 (2.9)	3 (2.6)	3 (3.2)	
Yellow	3 (1.4)	1 (0.9)	2 (2.2)	
Marital status				0.072^‡^
Single	190 (90.0)	102 (87.9)	88 (92.6)	
Married/stable union	19 (9.0)	14 (12.1)	5 (5.3)	
Separated/Divorced	2 (0.9)	0 (0.0)	2 (2.1)	
Education (years of schooling)	9.2±2.1	8,7±2.1	9,7±2.0	0.001^†^
Incomplete Elementary	115 (54.5)	72 (62.1)	43 (45.3)	
Complete Elementary	2 (0.9)	2 (1.7)	0 (0.0)	
Incomplete High School	92 (43.6)	40 (34.5)	52(54.7)	
Complete High School	1 (0.5)	1 (0.9)	0 (0.0)	
Incomplete Higher Education	1 (0.5)	1 (0.9)	0 (0.0)	
Family income^§^	954 (954-1483)	954 (954-1406)	954 (954-1912)	0.111^||^
Type of housing as for material				0.120^‡^
Masonry	117 (55.5)	57 (49.1)	60 (63.2)	
Mixed: wood and masonry	56 (26.5)	35 (30.2)	21 (22.1)	
Mixed: wood and tin	9 (4.3)	4 (3.4)	5 (5.3)	
Wood	29 (13.7)	20 (17.2)	9 (9.5)	
Type of housing as for porperty				0.062^‡^
Rent	3 (1.4)	0 (0.0)	3 (3.2)	
Financed	1 (0.5)	0 (0.0)	1 (1.1)	
Live in without cost	18 (8.5)	7 (6.0)	11 (11.6)	
Own	189 (89.6)	109 (94.0)	80 (84.2)	

* Variables described as mean ±standard deviation, median (25-75 percentiles) or n (%);

† Student's t test;

‡ Pearson chi-square test;

§ minimum wage R$ 954.00, Brazil, 2018;

|| Mann-Whitney test

In the bivariate analysis of sociodemographic data and working environment conditions of children and youth, it was found that the variables (age, gender, color, marital status, family income, type of housing as for material, type of housing as for property) did not present statistical significance (p>0.05) with occupational accidents (Table 1).

In contrast, regarding education (p=0.001) (Table 1), leisure activities such as riding a motorcycle (p=0.034) and listening to music (p=0.002) (Table 2), fish/shrimp as a result of work activity (p=0.039), daily working hours (p=0.010) (Table 3) and use of PPE (p<0.001), especially boots, waterproof gloves, straw hat and sunscreen (Table 4), statistical significance with work accidents of children and youth in the rural environment was found. Thus, those who had work accidents were less educated, rode motorcycles more often, listened to music less frequently, worked in agriculture, had fish/shrimp as a result of work, had increased daily workload and used boots, waterproof gloves, straw hat and sunscreen as PPE.

**Table 2:** Association of the leisure activities variables in a study with work accidents (n=211). South Region, Brazil, 2018

Variables*	Total Sample (n=211)	With occupational accident (n=116)	Without occupational accident (n=95)	P value
Having leisure activities	208 (98.6)	115 (99.1)	93 (97.9)	0.589^†^
Type of leisure activities^‡^				
Play ball/soccer	86 (41.0)	47 (40.9)	39 (41.1)	1.000^§^
Ride a bike	39 (18.5)	23 (19.8)	16 (16.8)	0.706^§^
Go out with friends	33 (15.6)	21 (18.1)	12 (12.6)	0.369^§^
Ride a motorcycle	14 (6.6)	12 (10.3)	2 (2.1)	0.034^§^
Swim in the lake	22 (10.4)	8 (6.9)	14 (14.7)	0.104^§^
Play video game	27 (12.8)	12 (10.3)	15 (15.8)	0.332^§^
Watch television	50 (23.7)	26 (22.4)	24 (25.3)	0.748^§^
Listen to music	15 (7.1)	2 (1.7)	13 (13.7)	0.002^§^
Ride a horse	19 (9.0)	11 (9.5)	8 (8.4)	0.979^§^
Leisure hours/day	4 (3-5)	4 (2-5)	4 (3-5)	0.169^||^

* Variables described as mean ±standard deviation, median (25-75 percentiles) or n (%);

† Fisher's exact test;

‡ with at least ten cases;

§ Pearson's chi-square test;

|| Mann-Whitney test

**Table 3:** Association of the variables activities at work, main products resulting from work and working time with occupational accidents (n=211). South Region, Brazil, 2018

Variables*	Total Sample (n=211)	With occupational accident (n=116)	Without occupational accident (n=95)	P value
Activities at work				
Fishing	78 (37.0)	50 (43.1)	28 (29.5)	0.058^†^
Crop planting	19 (9.0)	14 (12.1)	5 (5.3)	0.140^†^
Harvesting	20 (9.5)	15 (12.9)	5 (5.3)	0.098^†^
Main products resulting from work				
Lettuce	5 (2.4)	5 (4.3)	0 (0.0)	0.066^‡^
Tomato	9 (4.3)	7 (6.0)	2 (2.1)	0.190^‡^
Fish/Shrimp	84 (39.8)	54 (46.6)	30 (31.6)	0.039^†^
Working time (years)	3 (1-5)	3 (1-5)	2 (1-5)	0.375^§^
Daily working hours	4 (2-6)	4 (3-6)	4 (2-4)	0.010^§^

* Variables described as mean ±standard deviation, median (25-75 percentiles) or n (%);

† Pearson's chi-square test;

‡ Fisher's exact test;

§ Mann-Whitney test

**Table 4:** Association of the personal protective equipment variables in a study with occupational accidents (n=211). South Region, Brazil, 2018

Variables*	Total Sample (n=211)	With occupational accident (n=116)	Without occupational accident (n=95)	P value
Use of personal protective equipment	126 (59.7)	85 (73.3)	41 (43.2)	<0.001^†^
Boots	43 (20.4)	31 (26.7)	12 (12.6)	0.018^†^
Waterproof gloves	22 (10.4)	17 (14.7)	5 (5.3)	0.046^†^
Straw hat	25 (11.8)	21 (18.1)	4 (4.2)	0.004^†^
Sunscreen	53 (25.1)	39 (33.6)	14 (14.7)	0.003^†^
Raincoat	28 (13.3)	18 (15.5)	10 (10.5)	0.390^†^
Protective goggles	8 (3.8)	5 (4.3)	3 (3.2)	0.732^‡^
Jumpsuit	15 (7.1)	7 (6.0)	8 (8.4)	0.688^†^
Waterproof coat	4 (1.9)	2 (1.7)	2 (2.1)	1.000^‡^
Helmet	7 (3.3)	3 (2.6)	4 (4.2)	0.703^‡^
Arabic hat	4 (1.9)	4 (3.5)	0 (0.0)	0.128^‡^
Ear protector	3 (1.4)	1 (0.9)	2 (2.1)	0.589^‡^
Respirator	2 (0.9)	2 (1.7)	0 (0.0)	0.503^‡^
Visor	4 (1.9)	3 (2.6)	1 (1.1)	0.629^‡^
Water repellent pants	3 (1.4)	2 (1.7)	1 (1.1)	1.000^‡^
Reasons not to wear personal protective equipment				0.129^†^
The work environment does not offer danger	57 (86.4)	25 (89.3)	32 (84.2)	
The damage would occur anyway	1 (1.5)	0 (0.0)	1 (2.6)	
These are uncomfortable and uncomfortable gadgets	2 (3.0)	0 (0.0)	2 (5.3)	
Forget to use them	3 (4.5)	3 (10.7)	0 (0.0)	
High cost	2 (3.0)	0 (0.0)	2 (5.3)	
There is no supervision for use at work	1 (1.5)	0 (0.0)	1 (2.6)	

* Variables described as mean ±standard deviation, median (25-75 percentiles) or n (%);

† Pearson's chi-square test;

‡ Fisher's exact test

To control confounding factors, the variables that presented p<0.20 in the bivariate analysis were inserted in a multivariate Poisson regression model. Only variables with p<0.10 remained in the final model. After adjustment, they remained statistically associated with the occurrence of occupational accidents: type of housing as for material (p=0.047), leisure activities such as riding a motorcycle and swimming in the lake (p=0.001 and p=0.006, respectively), lettuce as a product resulting from work activity (p<0.001), use of PPE (p=0.015) and years of schooling (p=0.029) (Table 5).

**Table 5:** Factors independently associated* with the occurrence of occupational accidents in children and youth. South Region, Brazil, 2018

Variables	PR^†^ (CI 95%)^‡^	P value
Type of housing as for material		
Masonry	100	-
Mixed: wood and masonry	1.29 (1.00-1.66)	0.047
Mixed: wood and tin	0.70 (0.37-1.32)	0.269
Wood	1.24 (0.92-1.68)	0.160
Leisure activities		
Riding a motorcycle	1.69 (1.24-2.30)	0.001
Swimming in the lake	0.49 (0.29-0.82)	0.006
Listen to music	0.27 (0.07-1.02)	0.054
Product resulting from work activity (lettuce)	1.86 (1.34-2.59)	<0.001
Use of personal protective equipment	1.46 (1.08-1.97)	0.015
Years of schooling	0.95 (0.91-0.99)	0.029
Working time in the profession	1.03 (0.99-1.06)	0.087

* Poisson regression;

† PR = Prevalence Ratio;

‡ 95% CI = 95% confidence interval

Moreover, by Poisson regression, it was found that children and young people living in mixed housing (wood and masonry) were 29% more likely to have occupational accidents (PR=1.29; 95% CI: 1.00-1.66), when compared to those who lived in masonry housing. In addition, those riding a motorcycle showed an increase in the prevalence of occupational accidents by 69% (PR=1.69; 95% CI: 1.24-2.30). On the other hand, those who listened to music had a 51% reduction in the prevalence of these events (PR = 0.27; 95% CI: 0.07-1.02) (Table 5).

In addition, children and young people who had lettuce as a product resulting from work activities had an increased likelihood of occupational accidents of 86%, (PR=1.86; 95% CI: 1.34-2.59), being the use of PPE related to the increase in the occurrence of these events by 46% (PR=1.46; 95% CI: 1.08-1.97). Finally, for the increase of one year of education, there was a reduction in their prevalence by 5% (PR=0.95; 95% CI: 0.91-0.99).

## Discussion

The results showed that of the 211 respondents (children and youth), 116 (55%) were affected by accidents assisting their families in rural work. Accordingly, a study conducted in Canada^(^ 13 ^)^ identified a high incidence of occupational accidents in this same population, with annual rates of 15.8%. A larger proportion of these events were reported in low-income countries, as found studies such as Indian^(^ 14 ^)^, Chinese^(^ 15 ^)^, and Iranian^(^ 16 ^)^. These results reinforce occupational accidents, in this age group, as a public health problem because they indicate grievances that require interventions, in order to enable the improvement of quality of life in developed and developing countries.

In the Indian^(^ 14 ^)^, Iranian^(^ 16 ^)^, and Chinese^(^ 17 ^)^ studies, evidence of work-related injuries in children and young people assisting the family in rural work, animal events such as dog bites and insect bites , followed by falls and burns were found. These findings corroborate what was found in the present study and confirm the importance of identifying and acting on its causes.

In the case of this investigation, the occurrence of occupational accidents was associated with lack of attention at work (93.7%), which converged with the results of the North American research^(^ 18 ^)^. In this, it was found that children and young people who helped in agricultural work, over time, had reduced attention to activities they performed, favoring the occurrence of occupational accidents. Furthermore, it was pointed the need for training for them and their families on the risks to which they are exposed to, use of PPE, minimization of risk factors for health and well-being in the work environment and family monitoring while performing their activities.

Although this study did not identify the presence or absence of the relative at the time of the accident, other research found that two thirds of children killed in agricultural accidents were supervised by a relative and that in half of these events, the relative was close to them or provided to them exact instructions and information prior to the incident. Thus, it was found that family members often overestimate the skills of children who sometimes perform dangerous tasks, such as driving tractors and motor vehicles, fishing and caring for animals - horses, cows and others - which favors accidents^(^ 18 ^-^ 19 ^)^. Findings from the present study and those included in the argued dialogue reinforce the magnitude of a set of organized actions to meet the needs of the families of these children and youth. As a strategy, it is emphasized the need for training on risks and their management, which increases the ability to assess the existing hazard and the decision to avoid it.

It was also found that the most frequent occupational accidents in young people occurred at the age of 15 and among females (118; 55.9%). Although these variables are not statistically significant, they may constitute a risk factor for these circumstances. Despite the participants of this study, with a minimum sample representative of the population, it is understood that, even incited and informed about what is an accident, did not sufficiently understand what is an occupational accident. Accordingly, an Indian finding^(^ 19 ^)^ verified that occupational accidents in children and youth in the rural environment occurred more frequently between 6 and 15 years of age, but with a predominance of males. However, in another study^(^ 13 ^)^ there were high rates of these accidents in the age group of 10 years or less, which may be justified by cultural issues, that is, by the intergenerational transmission of the family tradition of rural work to have difficulty identifying what an occupational accident is.

Regarding education, 72 (62.1%) participants who experienced an occupational accident had incomplete elementary school. Surveys conducted in North Carolina^(^ 18 ^)^, Brazil^(^ 20 ^)^, Nepal^(^ 21 ^)^, and Canada^(^ 22 ^)^ confirmed this finding by verifying that children and young people who assisted in agriculture generally had incomplete primary education due to the prioritization work and financial difficulties. On the other hand, the low educational level of the participants may be related to the fact that they assist family members in rural work for a long timeframe and in the opposite shift from school attendance, which can cause fatigue and consequent dropout.

Regarding the daily working hours, this study showed that children and young people who worked with family members for long hours were at higher risk for accidents. A similar finding in a survey in Peru (23) was verified, whose data revealed that of the 375 children and young people who assisted in rural work, 363 (97%) had already suffered some type of occupational accident and that 188 (51,2%) worked more than eight hours a day. Prolonged hours lead to physical and mental exhaustion which, in addition to contributing to occupational accidents, make these individuals use their free time to rest and replenish their energy to continue the work the next day, having shorter time for recreational activities.

It was found that the participants had reduced time for leisure, which contributes to personal dissatisfaction, demotivation, decreased quality of life and, consequently, the occurrence of work accidents. In this sense, the literature points that young people who work in the rural environment spend many hours in their workplace and, because of this, feel overloaded and too tired for recreational activities, intensifying occupational injuries^(^ 24 ^)^. However, in a Chinese study, it was evidenced that individuals who can balance work and leisure hours self-report better vital energy indexes, motivation for daily activities, improved cognitive and emotional functions to manage stress and decrease the incidence of occupational accidents, compared to those who have no recreation or have little time for entertainment^(^ 25 ^)^.

It was also found that the participants who worked in fishing for fish and/or shrimp had a higher risk for occupational accidents. Similar data were checked in surveys conducted in Alaska^(^ 26 ^)^ and Canada^(^ 27 ^)^, in which children and young people who have fishing as work activity are subject to injury from sunburn and the handling of work tools such as knives and hooks and ciguatera (food poisoning). These evidences infer that the interaction between work and accidents and/or illnesses should not be neglected when planning health interventions with this public.

In addition, in the present study, the type of mixed housing showed a 29% higher probability of occupational accidents. In a study conducted in China^(^ 17 ^)^, it was verified that the material which the house is made of, can increase vulnerability to occupational accidents, meeting the findings of this study, in which there were higher rates of these occurrences in children and youth living in houses built from masonry and wood. It is unclear how this relationship occurs, however, it is believed that it may be associated with the low availability of financial resources of the family and as a consequence its use for the improvement and supplying the household instead of acquisition of protective equipment, which can indirectly increase the chances of occupational accidents.

Another relevant aspect evidenced by this study was the fact that children and young people who used motorcycles presented a 69% increase in the prevalence of occupational accidents. Its use as means of transportation by the participants is justified because it promotes easy mobility in the rural work environment due to the long distances traveled. A Canadian study^(^ 13 ^)^ showed that the use auto motors as work tools, such as tractors, pickup trucks, trucks and motorcycles, although facilitating the transportation of objects and loads in rural areas, doubled the incidence of accidents in children and young workers.

On the other hand, those who had leisure activities, such as listening to music, had a 51% reduction in the prevalence of occupational accidents. In this sense, the literature has revealed that recreational activities such as walking, cycling, yoga, music, meditation and participation in social groups, as well as school activities, impact the quality of life of children and young people and, as an effect, their ability to work^(^ 28 ^)^. Therefore, it is relevant to emphasize the need to keep a balance between work, recreation and school, due to the increase of technical-scientific knowledge about prevention and expansion of the ability to focus attention on work activities.

In the case of the present study, among the set of products resulting from the work activity of children and young people, only the activity related to lettuce crop presented the highest probability (86%) of occupational accidents. In this sense, the literature points that agricultural accidents, regardless of the product of the activity, were mainly related to the use of pesticides/agrochemicals^(^ 29 ^-^ 30 ^)^. Although this study did not address issues related to the use of pesticides, other research conducted in China found that the use of these products on crops, such as lettuce crops, associated with the use of sharp instruments such as knives, machetes and sickles, can cause, among other effects, the reduction of muscle strength and marching instability, which favors accidents related to cuts, falls and fractures^(^ 27 ^)^.

It is also noteworthy that the participants who used PPE, such as boots, waterproof gloves, straw hat and sunscreen, presented an increase of 46% in the occurrence of accidents in the rural work, which may decrease this probability by 5% with the increase of one year of schooling. This finding is consistent with an integrative review study that found that children and young people engaged in agricultural and fishing activities, with low education, used less PPE, or used it inappropriately or incompletely, compared to students with more education^(^ 24 ^)^. This are findings that may suggest the need to encourage the use of PPE and training for proper handling of this equipment, in places easily accessible to this clientele, such as schools, basic health units and family homes. Examples include sunscreen and replacement at the correct time, according to the manufacturer of the product, as well as clothing such as jumpsuits, T-shirts, shoes, gloves and adequate hats that protect the body from exposure to accidental and health hazards.

As a limitation of the study, it is emphasized the exploratory nature that, despite having enabled the achievement of the proposed objectives, did not allow acting on the identified scenario. In addition, we highlight as weakness the use of a single subjective source of data - self-reference. However, this becomes necessary for the situational diagnosis of rural work as a causal nexus of accidents, since it is the product of self-observation and self-reflection of the individual about their work experiences, being central for the planning and development of educational-care actions. Moreover, there is a lack of publications on this subject, especially action research/intervention research.

With the results of this study, there is progress in knowledge by showing, through multivariate analysis tests, the cause-and-effect relationship between rural work assistance and the occurrence of occupational accidents in children and youth. Therefore, it can subsidize interventions through risk communication, which will enable the expansion of knowledge about these accidents in this clientele.

## Conclusion

In the present study it was found that the prevalence of occupational accidents such as insect bites, burns and falls in the workplace, in children and young people who worked with their families in the rural environment was expressive and was related to the type housing, leisure activities such as riding a motorcycle, product resulting from work activity, such as lettuce cultivation and use of PPE. On the contrary, it was evidenced that leisure activities such as listening to music and the addition of one year of schooling contribute to the reduction of work accidents. It is believed that these findings may increase the development of social policies aimed at preserving the health of this public, controlling working conditions and reducing occupational risks in the rural environment.

In addition, the identification of the most prevalent occupational accidents and their associated factors may support actions in schools and in traditional primary health care and family health strategy units, with a view to equipping these children and youth to develop their work activities in the rural environment free of harm to their health and education. The nurse stands out with an important role in this process. He/she uses knowledge to understand the clinical characteristics of these individuals, the working conditions and occupational environments to which they are exposed and that aim to direct health interventions to this age group. Thus, it will assist in collective health surveillance, through the exploration and dissemination of pertinent information about the work environments and the health of children and young people who assist the family in rural work, as well as training on the correct use and handling of protective equipment and appropriate body mechanics to the activities performed.
